# A Strategy Matching Tool for Boosting Implementation of Geriatric Telehealth Services in Rural CBOCs

**DOI:** 10.1093/geroni/igab046.458

**Published:** 2021-12-17

**Authors:** Laura Kernan, Eileen Dryden, Camilla Pimentel, Kathryn Nearing, Lauren Moo

**Affiliations:** 1 Veterans Health Administration, Bedford, Massachusetts, United States; 2 VA Bedford Healthcare System, Bedford, Massachusetts, United States; 3 Veterans Health Administration, Denver, Colorado, United States; 4 VA Bedford Health Care System, Bedford, Massachusetts, United States

## Abstract

Fifteen Veterans Administration Medical Centers (VAMCs) offer geriatric specialty care telehealth services through a hub and spoke model to patients at affiliated community-based outpatient clinics (CBOCs). These services are not used to the extent they could be. Through interviews with 50 staff and providers at rural CBOCs we identified several implementation facilitators and barriers. CBOC-level barriers included space constraints, low staffing, internet connection issues, and limited knowledge of services available and referral processes. Patient-level barriers included discomfort with technology, cognitive decline, and inability to travel to the CBOC. We found that champions within the CBOC and iterative, targeted outreach from the hub helped facilitate uptake of services. We entered the identified barriers into the CFIR-ERIC (Consolidated Framework for Implementation Research-Expert Recommendations for Implementing Change) Implementation Strategy Matching Tool to help generate targeted strategies that will be used to refine each hub’s implementation approach.

